# The Impact of Dialysis-Requiring Acute Kidney Injury on Long-Term Prognosis of Patients Requiring Prolonged Mechanical Ventilation: Nationwide Population-Based Study

**DOI:** 10.1371/journal.pone.0050675

**Published:** 2012-12-12

**Authors:** Chia-Ter Chao, Chun-Cheng Hou, Vin-Cent Wu, Hsin-Ming Lu, Cheng-Yi Wang, Likwang Chen, Tze-Wah Kao

**Affiliations:** 1 Division of Nephrology, Department of Internal Medicine, National Taiwan University Hospital, Taipei, Taiwan; 2 Department of Internal medicine, Min-Sheng General Hospital, Tao-Yuan, Taiwan; 3 Institute of Population Health Sciences, National Health Research Institutes, Zhunan, Taiwan; 4 Department of Internal Medicine, Cardinal Tien Hospital, Xindian, Taiwan; 5 School of Medicine, Taipei Medical University, Taipei, Taiwan; D'or Institute of Research and Education, Brazil

## Abstract

**Background:**

Prolonged mechanical ventilation (PMV) is increasingly common worldwide, consuming enormous healthcare resources. Factors that modify PMV outcome are still obscure.

**Methods:**

We selected patients without preceding mechanical ventilation within the one past year and who developed PMV during index admission in Taiwan's National Health Insurance (NHI) system during 1998–2007 for comparison of mortality and resource use. They were divided into three groups: (1) patients with end-stage renal diseases (ESRD) before the index admission for PMV onset; (2) patients with dialysis-requiring acute kidney injury (AKI-dialysis) during the hospitalization course; and (3) patients without AKI or with non dialysis-requiring AKI during the hospitalization course (non-AKI). We used a random-effects logistic regression model to identify factors associated with mortality.

**Results:**

Compared with the other two groups, patients with AKI-dialysis had significantly longer mechanical ventilation, more frequent use of vasopressors, longer intensive care unit/hospital stay and higher inpatient expenditures during the index admission. Relative to non-AKI patients, patients with AKI-dialysis had an elevated mortality hazard; the adjusted relative risk ratios were 1.51 (95% confidence interval [CI]:1.46–1.56), 1.27 (95% CI: 1.23–1.32), and 1.10 (95% CI: 1.08–1.12) for mortality rates at discharge, 3 months, and 4 years after PMV, respectively. Patients with AKI-dialysis also consumed significantly higher total in-patient expenditure than the other two patient groups (p<0.001).

**Conclusions:**

Among patients that need PMV care during an admission, the presence of de novo AKI requiring dialysis significantly increased short and long term mortality, and demand for health care resources.

## Introduction

Prolonged mechanical ventilation (PMV), defined as continuous receipt of artificial respiratory support for at least 21 days [1], has gained growing attention due to population aging and substantive improvement in acute care [2], [3]. Patients surviving their initial critical illness frequently end up with residual multi-organ dysfunction, leading to subsequent PMV [4]. In light of the enormous economic impact of PMV, effort has been increasingly devoted to identifying the factors associated with the development and outcome of PMV [5].

Previous reports indicate that acute kidney injury (AKI) significantly worsens hospitalization outcomes, reduces weaning probability, and portends significantly poorer long-term prognosis [6]–[8]. Patients developing AKI during admission frequently have multiple comorbidities (diabetes mellitus, cerebrovascular events, pre-existing renal insufficiency), and also have increased transfusion requirement, need of repeated procedures and higher risk of subsequent chronic kidney disease development [9]–[12]. Even transient AKI exerts an unfavorable effect on long-term outcome comparing with persistent AKI [13]. Similarly, factors such as fluid overload and AKI at the start of ventilation or during subacute respiratory care are associated with PMV development in intensive care units (ICUs) [14]–[16], while receipt of renal replacement therapy (RRT) may predict short-term mortality in patients requiring PMV [17]–[19]. Consequently renal failure plays a vital role over the courses of PMV development and outcome [14]–[16], [20].

Literature is sparse regarding the impact of AKI, especially dialysis-requiring one, on the long-term prognosis of patients requiring PMV. In addition, there is evidence that PMV patients might behave differently from the entire critical care population [19]. Carson et al, in their single-center experience, demonstrated that severity-of-illness indexes such as APACHE (acute physiology and chronic health evaluation) scores failed to predict mortality in patients with prolonged critical illnesses [21]. Several studies also delineated the physiologic distinctions in PMV population contrary to the ICU residents, including the prolonged influence of barotrauma, the pace of weaning, and also the differences in staffing [22]–[24]. In light of the enormous economic burden and poorer prognosis stemming from PMV [25], it is therefore vital to specifically focus on PMV patients with regard to their outcome determinants.

We hypothesized that dialysis-requiring AKI occurring during hospitalization with subsequent PMV development may worsen the long-term prognosis, in addition to short-term outcomes. Using data from a nationally representative sample of patients receiving PMV in Taiwan [3], we compared patient-level resource use and overall prognosis among PMV patients with different degrees of renal function during their hospital stay.

## Methods

### Study design and setting

This retrospective cohort study was a secondary analysis of the prospectively collected data in the Taiwan's National Health Insurance (NHI) and Household Registration databases. The research project was approved by the National Health Research Institutes (NHRI); all individual identification numbers were scrambled by the Bureau of National Health Insurance (BNHI) for privacy protection, and specific patient consent was deemed unnecessary by the institutional review board. As almost 99% of Taiwanese are covered by the NHI and they fully utilize all NHI medical services, the NHI system is an appropriate research setting for this study.

### Original data sources

Two government organizations of Taiwan, the BNHI and the Ministry of the Interior, supplied the original research data. The NHI database by the BNHI contains detailed information of all insured persons including health services, procedures and prescriptions they received as well as their background conditions and diagnoses. The diagnostic coding is based on the International Classification of Diseases, Ninth Revision (ICD-9). As the BNHI routinely audits claims data to prevent fraud in the NHI program, the NHI data is generally reliable [26]. The death certificate data were retrieved from the well-maintained Household Registration database by the Ministry of the Interior. All of our study subjects with PMV had well-managed records in the household registration system.

### The PMV database and patient cohorts

The NHRI processed the original data and constructed a longitudinal database for those patients 17 years of age or older at the PMV onset in 1998–2007 [3]. To focus on new PMV incidences, the NHRI only included the first PMV incidence before which the patient had not used invasive ventilators, negative pressure ventilators or positive pressure ventilators for at least one year.

We excluded from the PMV population patients who already received kidney transplant and patients with pre-admission AKI or dialysis within a 6-month period prior to the index hospital stay. Those requiring permanent dialysis before the index admission were separately identified. The following three cohorts were then formed: (1) ESRD patients requiring permanent dialysis before the index admission for the PMV onset (“the ESRD cohort”); (2) patients with de novo dialysis-requiring AKI during hospitalization (“the AKI-dialysis cohort”); and (3) patients without AKI or with non dialysis-requiring AKI during hospitalization (“the non-AKI cohort”). Detailed definitions for PMV and AKI, as well as a figure that depicts the PMV database construction procedures and the cohort selection process, were presented in Figure S1 and the appendix file. We followed each patient until death or the end of 2007.

### Research variables and endpoints

We used SAS software version 9.1.3 (SAS Institute Inc., Cary, NC) to extract data from the PMV database. Epidemiologic data collected in this study included patient age and gender, route of admission to ICU (through emergency department [ED] or not), and organ dysfunctions developed during the index admission. Patient comorbidities, types of major operation received, number of days hospitalized, number of chest films taken and number of outpatient visits due to lung diseases within the year prior to the index admission were also recorded. To determine pre-existing comorbidities, we used a relatively strict criterion: at least one inpatient admission or at least three outpatient visits for treating the disease during the year prior to the index admission is needed. Our primary endpoints were the in-hospital mortality as well as the mortalities at 3 months, and 4 years after the development of PMV.

Our secondary endpoints were the in-hospital mortality at 6 months, 1 year, 2 years and 3 years after PMV onset, the resource use and expenditures during the index hospital stay with PMV; these parameters included the frequency of vasopressor use, length of MV, length of dialysis, duration of ICU stay and duration of the index hospitalization. The total NHI inpatient expenditures were also recorded according to reimbursement data in the NHI, being true costs. Our main analysis focused on comparison between patients with AKI-dialysis in the index admission and their non-AKI counterparts (the control group). For sensitivity analysis, we also compared patients with AKI-dialysis and those with ESRD.

### Statistical analysis

We used Stata software version 9 (StataCorp, College Station, TX) to perform descriptive and multivariate regression analyses. Continuous variables are described as mean ± standard deviation (SD) while discrete variables are presented as counts or percentages. We adopted a random-effects logistic regression model to identify factors associated with post-PMV mortality in PMV patients (see Table S1 for more detailed description of the method). We further employed the logistic regression method to estimate the prognostic models separately for the primary outcomes (in-hospital, 3-month, 4-year post-PMV mortality rates) and the secondary outcomes (6-month, 1-year, 2-year, 3-year post-PMV mortality rates).

Using a significant level of 5%, we reported adjusted RRRs for all explanatory variables and mortality models (for different lengths of follow-up time). A stepwise procedure was used to choose explanatory variables that were suitable for stepwise selection (such as a continuous variable, or a dummy variable alone for a specific feature), and we decided to include all explanatory variables in each model. Results from the logistic regression by reporting the adjusted odds ratios (ORs) of explanatory variables and their 95% confidence intervals (CIs) were available in the supplementary files (Tables S1 and S2). To take unobserved heterogeneity among hospital-year clusters into account, a random-effects model was employed to control the shared frailty within each cluster. By means of a previously reported method, we converted estimates of ORs into estimates of relative risk ratios (RRRs); the latter estimates can better reflect the influences of covariates on the likelihood of incidence of an event [27]. All ORs and RRRs were adjusted for aforementioned epidemiologic variables.

## Results

### Demographic and clinical characteristics of patients under PMV

As shown in Table 1, non-AKI patients, the oldest (72.5±14.9 years) group, had the highest proportion of males (61.6%), whereas ESRD patients, the youngest (69.5±11.2 years), had the lowest proportion of males (47.2%) (all comparisons with p<0.001). Cardiovascular and pulmonary comorbidities were found in over 50% of the patients in all three groups. Hypertension, diabetes and neurological diseases were also prevalent, with a graded increase from non-AKI patients, patients with AKI-dialysis to ESRD patients.

**Table 1:** Demographic and clinical characteristics of patients under PMV.

	ESRD prior to PMV (n = 1,015)	AKI-dialysis during the index admission (n = 5,129)	Non-AKI during the index admission (n = 41,610)	p-value: trend†
*Gender*				
Male (%)	47.2	54.5	61.6	<.001
*Age in years* (mean [SD])	69.5 (11.2)	71.9 (13.8)	72.5 (14.9)	<.001
*Age group*				
<45 (%)	2.9	5.5	6.6	<.001
45–64 (%)	28.0	18.3	16.3	
65–74 (%)	34.2	28.0	23.4	
>74 (%)	35.0	48.2	53.6	
*ED admission* (%)	53.3	60.2	58.2	0.001
*ICU admission (%)*	97.0	98.0	92.6	<.001
*Organ dysfunctions during the index admission (excluding lungs and kidneys)*				
Cardiovascular (%)	24.9	22.3	15.6	<.001
Hepatic (%)	1.3	1.9	1.3	0.002
Neurologic (%)	9.1	5.0	5.4	<.001
Hematologic (%)	1.7	2.6	1.3	<.001
Metabolic (%)	0.5	1.3	0.5	<.001
*Number of organ dysfunctions during the index admission (excluding lungs and kidneys)*				
0 (%)	66.2	69.6	77.7	<.001
1 (%)	30.3	27.7	20.5	
2 (%)	3.5	2.6	1.7	
3 (%)	0.1	0.0	0.1	
4+ (%)	0.0	0.0	0.0	
*Comorbidity in the year prior to the index admission*				
Parkinson's disease (%)	1.3	2.0	2.7	<.001
MS or degenerative nervous system (%)	1.2	1.4	1.9	0.027
Neurologic (%)	39.7	34.9	34.3	0.001
Cardiovascular (%)	85.1	71.9	64.9	<.001
Pulmonary (%)	58.7	57.0	63.9	<.001
COPD (%)	4.8	9.7	18.8	<.001
Renal (%)	100.0	42.7	29.3	<.001
Hepatic (%)	12.8	11.2	9.7	<.001
Cancer (%)	9.2	11.1	12.9	<.001
Diabetes (%)	58.4	41.7	24.3	<.001
Hypertension (%)	69.0	57.1	45.5	<.001
*Charlson index* (mean [SD])	4.4 (2.0)	2.6 (2.3)	2.1 (2.3)	<.001
*Major operations during the year prior to the index admission*				
Cardiac/thoracic aorta (%)	4.0	1.7	1.7	<.001
Liver/bililary/pancreas (%)	2.9	2.6	2.3	0.200
Lower digestive tract (%)	2.3	2.5	1.9	0.013
Upper digestive tract (%)	1.7	2.0	1.8	0.504
*Number of days with inpatient care during the year prior to the index admission* (mean [SD])	41.7 (48.2)	18.6 (32.1)	23.9 (42.5)	<.001
*Number of chest films taken during the year prior to the index admission* (mean [SD])	0.02 (0.16)	0.02 (0.18)	0.03 (0.22)	<.001
*Number of outpatient visits due to lung diseases during the year prior to the index admission* (mean [SD])	3.6 (5.7)	5.8 (8.9)	7.7 (11.1)	<.001
*In-hospital mortality (%)*	47.4	59.9	34.5	<.001

† Chi-Square test for percentages; ANOVA for means.

Abbreviations: AKI, acute kidney injury; COPD, chronic obstructive pulmonary disease; ED, emergency department; ESRD, end-stage renal disease; ICU, intensive care unit; MS, multiple sclerosis; PMV, prolonged mechanical ventilation; RRT, renal replacement therapy; SD, standard deviation.

ESRD patients had the highest Carlson index and were more likely to receive major cardiothoracic operation during the year before the index admission (4.0%, versus 1.7% for both Groups 2 and 3; both p<0.001), indicating that they might be sicker than the others during the year before the index admission. ESRD patients also had the longest hospital stay during the year before the index admission (41.7 days, versus 18.6 days for patients with AKI-dialysis and 23.9 days for non-AKI group; both p<0.001). On the contrary, non-AKI patients visited the outpatient clinic for pulmonary diseases more frequently than the other two groups during the year before the index admission (7.7 visits, versus 3.6 visits for ESRD patients and 5.8 visits for patients with AKI-dialysis; both p<0.001).

Patients with AKI-dialysis were most likely to be admitted through the EDs (60.2%) and also most likely to be admitted directly into the ICUs (98.0%). Moreover, the hospitalization outcome was also worst in patients with AKI-dialysis, with the highest in-hospital mortality (59.9%, versus 47.4% for ESRD patients and 34.5% for those with no AKI or non dialysis-requiring AKI; both p<0.001). During the index admission, cardiovascular system was the most commonly failed organ system in both ESRD patients and those with AKI-dialysis (24.9% and 22.3% respectively), while neurologic system ranked the second (9.1% in ESRD patients; 5.0% in patients with AKI-dialysis; and 5.4% in non-AKI patients).

### Resource use and expenditures during the index hospital stay with PMV

As shown in Table 2, patients with AKI-dialysis consumed significantly more resources during the index admission. It had the longest duration of MV use (62.2 days, versus 53.4 days for ESRD patients and 58.7 days for non-AKI patients; both p<0.05), longest ICU stay (40.1 days, versus 32.0 days for ESRD patients and 27.3 days for non-AKI patients; both p<0.001), and longest hospital stay (81.1 days, versus 73.0 days for ESRD patients and 75.4 days for non-AKI patients; both p<0.01). The average total inpatient expenditures for patients with AKI-dialysis during the admission were 984,665 New Taiwan Dollars (TWD) - approximately 33,000 US dollars, significantly higher than those for ESRD patients (827,008 TWD; p<0.001) or non-AKI patients (651,606 TWD; p<0.001).

**Table 2:** Resource use and expenditures during the index hospital stay with PMV.

	ESRD prior to PMV (n = 1,015)	AKI-dialysis during the index admission (n = 5,129)	Non-AKI during the index admission (n = 41,610)	p-value: trend†
*Vasopressor use* (%)	50.3	50.1	29.6	<.001
*Mechanic ventilation (days)* (mean [SD])	53.4 (57.4)	62.2 (88.2)	58.7 (103.2)	0.015
*Renal replacement therapy (days)* (mean [SD])	36.8 (48.5)	15.2 (25.1)	0.0 (0.0)	<.001
*ICU stay (days)* (mean [SD])	32.0 (22.3)	40.1 (29.1)	27.3 (18.8)	<.001
*Hospital stay (days)* (mean [SD])	73.0 (64.6)	81.1 (93.6)	75.4 (107.4)	0.001
*Inpatient expenditures (NTD)* (mean [SD])	827,008 (571,035)	984,665 (719,107)	651,606 (531,758)	<.001

† Chi-Square test for percentages; ANOVA for means.

Abbreviations: AKI, acute kidney injury; ESRD, end-stage renal disease; ICU, intensive care unit; NTD, New Taiwan Dollar (of 2010); PMV, prolonged mechanical ventilation; SD, standard deviation.

### Relative risk of death

As shown in Tables S1 and S2, factors associated with post-PMV mortality included renal status during the index admission, age, organ dysfunction, and cancer as a comorbidity. Advanced age and male patients had a higher mortality hazard (p<0.001). Initial ICU admission was also associated with higher mortality at 3 months after PMV and later on (p<0.001). PMV patients who had 3 or more failing organs in addition to lungs and kidneys would have an increase in in-hospital mortality by 60%, by comparison with those without organ failure (p<0.001). The presence of cancer increased in-hospital mortality as well as 3-month post-PMV mortality by 37% and worsened the outcome significantly (p<0.001). Hepatic disease also elevated mortality risk at discharge or 3 months after PMV.

Relative to non-AKI patients, patients with AKI-dialysis had an elevated mortality hazard (adjusted RRR = 1.51, 95% confidence interval [CI]: 1.46–1.56; 1.27, 95% CI: 1.23–1.32; and 1.10, 95% CI: 1.08–1.12, for mortalities at discharge, 3 months, and 4 years after PMV, respectively). Although impacts of the above-mentioned risk factors (i.e. renal status during the index admission with PMV onset, age, organ dysfunction and cancer as a comorbidity) diminished over time, they remained influential even at 4 years after PMV (Table S2). Furthermore, it should be noted that dialysis-requiring AKI during an admission with new onset of PMV might have a long-lasting harmful effect on life.

## Discussion

After adjustment for multiple confounders, we identified a significant correlation between the renal function status during admission and the long-term survival of patients under PMV. Comparing with non-AKI patients with PMV, patients with dialysis-requiring AKI during an admission with PMV onset had worse prognosis, longer hospital stay and higher demand for healthcare resources. In addition, our sensitivity analysis revealed that dialysis-requiring AKI also predicted worse outcomes, compared to pre-admission ESRD.

Reportedly, ICU patients under PMV are more likely to become resource intensive and tend to have higher post-discharge mortality than other ICU survivors [28]–[31]. The rationale for the poorer outcome among survivors under PMV may stem from a heavier comorbidity burden, a higher degree of functional dependency after critical illness, and a larger proportion of elderly people [18], [30], [31]. Increased frequency of pulmonary infection and PMV-induced mechanical lung injury may also play an important role in the higher mortality among PMV patients.

Statistics showed that about 50% of the PMV patients in Taiwan died within 3 months after the PMV onset, and the 1- and 4-year post-PMV mortality rates were around 70% and 80% respectively [32]. Data in our current study indicated that PMV patients with ESRD or dialysis-requiring AKI had particularly high risk of death before discharge (47% for ESRD, 60% for AKI; Table 1), and a substantial proportion of PMV patients discharged alive would pass away within a few years after discharge. Such phenomenon highlights the need for more research on factors increasing the mortality in PMV patients.

In population-based studies, dialysis-requiring and non dialysis-requiring AKI reportedly predict higher mortality and increased risk of progressive renal failure [6]–[8], [33], [34]. However, very little is known regarding the differences in outcome between patients with AKI and those with pre-existing ESRD in PMV patients [35]. Several small studies find that AKI in ICU patients correlated with worse outcome when compared with ESRD [36]–[38]. Consistent with their findings, we further showed that dialysis-requiring AKI in patients with PMV portends excess both short-term and long-term mortality compared with patients with ESRD (Table S2). The compound effect of MV on prognosis might account for this difference [39]. Consequently, dialysis-requiring AKI is much more harmful than ESRD in patients with PMV - a more vulnerable population of ICU patients.

AKI occurring during MV might have other detrimental effect. Vieira et al have found that a more than 85% increase in serum creatinine and oliguria during ICU stay are associated with a greater than 2-fold risk of weaning failure [40]. The positive fluid balance, disturbed acid-base status with increased work of breathing and the systemic inflammation accompanying kidney shut-down might contribute to the prolonged requirement of MV. In addition, occurrence of AKI in ICUs is associated with greater hemodynamic instability, higher incidence of leukocytosis and increased extra-renal organ failure [36], [41], [42], facilitating the inexorable clinical deterioration. Consequently, ICU patients with AKI have more organ failures and longer ICU stays than those with ESRD [43]–[46], consistent with our findings. On the contrary, ICU patients with ESRD have more comorbidities than their non-ESRD counterparts, and several studies reported that although ESRD and non-ESRD patients might have similar short-term outcomes, long-term prognosis was worse in the former. [43], [44]. In this sense, comparison of outcomes between PMV patients with de novo AKI and those with pre-existing ESRD is further complicated by patient comorbidities and clinical courses. The present study may shed light on this important issue.

**Figure 1:**
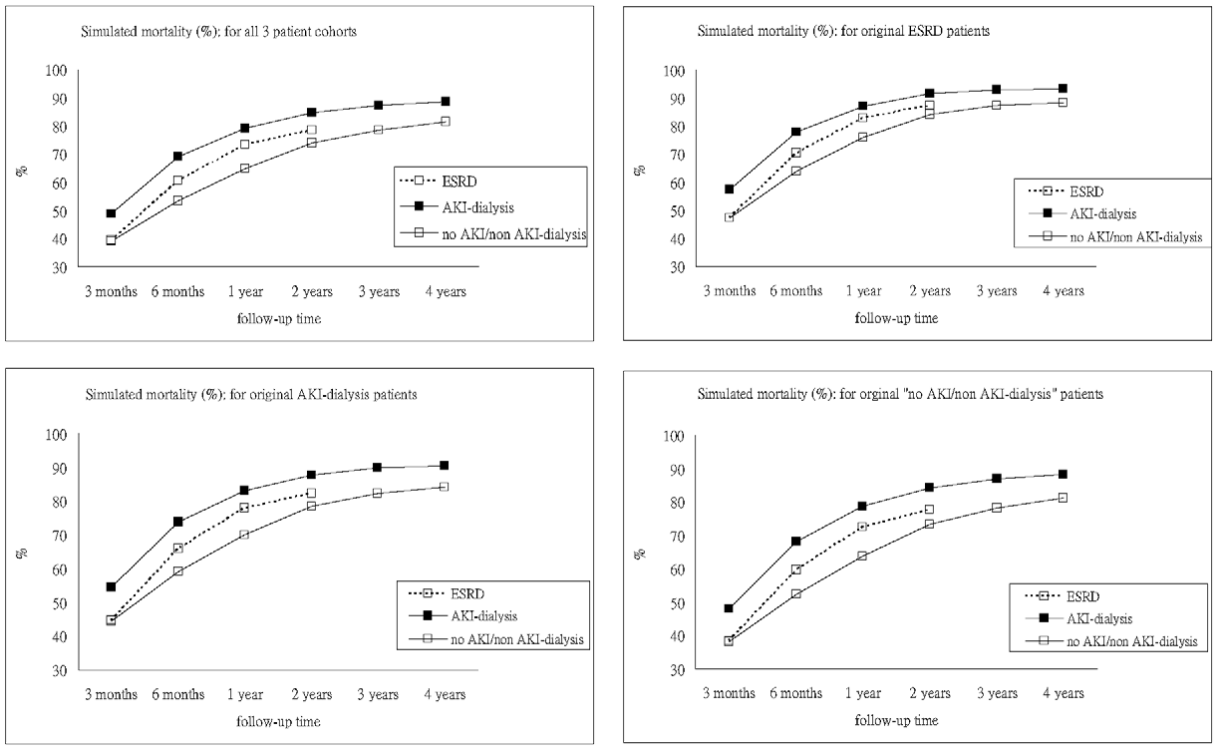
Mortality rates corresponding to different renal statuses: simulated results with all other covariates kept at the original values (no AKI/non AKI-dialysis: patients with no AKI or non dialysis-requiring AKI). Abbreviations: AKI, acute kidney injury; ESRD, end-stage renal disease.

This study also investigated how dialysis-requiring AKI occurring during an admission with PMV onset might influence long-term mortality. Comparing ESRD group and dialysis-requiring AKI group, we found that the excess risk of mortality from dialysis-requiring AKI during hospitalization decreased progressively to 23% and 12% at 3 month and 6 months after PMV onset respectively (Table S2). The excess risk further diminished to less than 10% at 1-year post-PMV but remained significant. The excess risk rose again after 4 years post-PMV. This resurgence of risk might be due to the heterogeneity between ESRD patients receiving PMV care in the early 2000s or earlier and ESRD patients under PMV at a later time. ESRD patients with higher frailty expectedly showed higher mortality, and the corresponding analysis would generate lower adjusted ORs for death when these patients were compared with AKI patients. This implies that the level of excess risk of death could vary with the inherent frailty of ESRD patients selected for comparison. We further conducted sensitivity analyses on the adjusted ORs and RRRs of 3-year and 4-year post-PMV mortalities under different levels of frailty among ESRD patients (Table S3). This phenomenon underlies the significance of checking inherent frailty among research study subjects in the outcome comparison between AKI and ESRD. Nonetheless, patients with dialysis-requiring AKI still fared poorer than patients with ESRD in all scenarios. Finally, we simulated the mortality rates with different renal statuses in the corresponding curves, as exhibited in Table S4 and Figure 1 respectively.

It is evident that PMV patients consume enormous healthcare resources [5], [25], [29], [47]. PMV patients with dialysis-requiring AKI are particularly resource-intensive. During the index admission with PMV, patients with dialysis-requiring AKI had healthcare expenditures (mean = 984,665 TWD; 2010) approximately 50% higher than it would be without AKI or with non dialysis-requiring AKI (mean = 651,606 TWD; 2010). It is thus vital to construct a comprehensive care plan for patients with PMV and dialysis-requiring AKI during hospitalization to minimize initial organ damages and allocate subsequent healthcare resources, including renal palliative care, judiciously [48].

Our study has certain strengths. It is a large population-based analysis of the factors correlating with PMV patient outcomes. A nationwide study design also largely reduced the effect of referral bias, which was often seen in critical care studies [49]. Furthermore, an extended follow-up period allowed us to determine the impact of each factor over the clinical course of PMV patients. However, our study has its limitations. First, the administrative database did not contain clinical information such as reasons for ICU admission, ICU types (surgical or medical), physiologic recordings (blood pressure, heart rate, etc.), severity scoring (acute physiologic and chronic health evaluation, APACHE), and pertinent laboratory parameters. Lacking these factors in the database inhibited us from further investigating how they might interact with AKI in affecting patients' prognosis [9], [11]. Nevertheless, analysis results from this large population database helps in affirming a phenomenon. That is, the presence of de novo AKI requiring dialysis carried significantly higher risk of short and long term mortality and larger demand for health care resources among a population of PMV patients, with wide variations in levels of aforementioned factors. Second, the way of recording the diagnosis of dialysis-requiring AKI did not permit us to further classify the patients according to the existing schemes, (RIFLE, or AKIN classifications) [50]. However, we believe that categorization of patients based on dialysis utilization might facilitate the spread of key messages among policy-makers and their application. Third, some medical facilities might over-report the diagnoses for reimbursement purposes. Fortunately, this confounding issue is likely minor, as the BNHI adopts strict criteria for reporting critical illnesses [3], and we used strict criteria in ascertaining the pre-existing comorbidity in this study.

In conclusion, AKI requiring dialytic support that occurred during an index admission with PMV care could bring about higher short and long-term risk of death as well as larger demand for healthcare resources than pre-admission ESRD. These findings shed light on the necessity to provide comprehensive care for hospitalized PMV patients with dialysis-requiring AKI and the importance of AKI prevention during PMV care.

## Supporting Information

Appendix S1 **Definitions for prolonged mechanical ventilation (PMV) and dialysis-requiring acute kidney injury (AKI).**
(DOC)

Figure S1 **Flow diagram of the selection process of the study objects.**
(TIFF)

Table S1 **Adjusted odds ratios – estimates based on random-effects logistic regression results for mortality.** Part 1: In-hospital, 3-month past PMV, and 6-month post-PMV mortality rates. Part 1A: In-hospital, 3-month past PMV, and 6-month post-PMV mortality rates: sensitivity analysis using the ESRD group as the reference group. Part 2A: 1-year, 2-year, 3-year and 4-year past PMV mortality rates: sensitivity analysis using the ESRD group prior to PMV as the reference group.
(DOC)

Table S2 **Adjusted relative risk ratios – estimates based on random-effects logistic regression results for mortality.** Part 1: Main analysis using patients without AKI-dialysis as the reference patient group. Part 2: sensitivity analysis using patients with ESRD as the reference patient group.
(DOC)

Table S3 **Sensitivity analysis on the adjusted RRRs of PMV patients with dialysis-requiring AKI for 3-year and 4-year mortality rates.**
(DOC)

Table S4 **Simulated mortality rates (%) for different renal statuses.**
(DOC)
