# Supplementary material for: The Impact of Dialysis-Requiring Acute Kidney Injury on Long-Term Prognosis of Patients Requiring Prolonged Mechanical Ventilation: Nationwide Population-Based Study
Source: PLoS One. 2012 Dec 12;7(12):e50675. doi: 10.1371/journal.pone.0050675 (PMC3520952; doi:10.1371/journal.pone.0050675)
Supplement: Appendix S1 — Definitions for prolonged mechanical ventilation (PMV) and dialysis-requiring acute kidney injury (AKI). (DOC) [file pone.0050675.s001.doc]

**Appendix – Definitions for prolonged mechanical ventilation (PMV) and dialysis-requiring acute kidney injury (AKI)**

The study setting was in the Taiwan National Health Insurance (NHI). The NHI care program for PMV covers invasive ventilators, negative pressure ventilators, and positive pressure ventilators, under the condition that at least some use of an invasive ventilator or a negative pressure ventilator should be made prior to the first day of using a positive pressure ventilator. Thus, PMV patients in the NHI only include those under MV care. Patients with a tracheostomy tube in situ not connected to a ventilator are not regarded as ventilator-dependent in the NHI. Additionally, this PMV program was designed for patients 17 years of age or older.

Based on requirements for the PMV care program in the NHI, we had the following criteria for selecting study patients: (1) continuous use of invasive ventilators, negative pressure ventilators, and/or positive pressure ventilators for at least 21 days; (2) use of invasive ventilators or negative pressure ventilators at the initiating stage of care; (3) >=17 years of age on the 21st day of mechanical ventilation. We also confined the patient cohort to those becoming under PMV in or after 1998, when extensive use of mechanical ventilation (MV) started to receive much attention in Taiwan. Finally, our study focused on PMV events that had no use of invasive ventilators, negative pressure ventilators, and positive pressure ventilators for at least one year immediately before the beginning of PMV. This was based on an idea that findings from investigating outcomes among “new” patients with PMV can provide more reference information to physicians and policy makers, compared with results from examining future outcomes among patients who have been using MV services continuously or intermittently for months or even years.

With regard to AKI, we identified AKI diagnosis using the following 3-digit ICD-9-CM (International Classification of Diseases, Ninth Revision, Clinical Modification) codes: 580, 581 and 584. This study focused on de novo advanced AKI. Within our study sample, except those with permanent need for dialysis (ESRD) before PMV care, all patients had neither AKI diagnosis nor dialysis for at least 6 months before PMV care. PMV patients defined as those with de novo dialysis-requiring AKI were “PMV patients using dialysis during the index hospitalization among non-ESRD patients within the study sample.”

We depicted the complete selection process of the study subjects in figure S1.

A sub-sample obtained after simple random sampling with a fraction of 1/(3.4) to reduce the sample size to 10% of the whole population (the upper limit of sample size for using National Health Insurance data)

(n=2,619,534; **10%** of all enrollees ever alive in the 12 years)

New patients with prolonged mechanical ventilation (PMV) in 1998-2007 (to focus on patients in or after 1998, when use of mechanical ventilation started to receive much attention)

(n=50,481)

Inclusion criteria:

1. Continuous use of invasive ventilators, negative pressure ventilators, and/or positive pressure ventilators for at least 21 days

2. Use of invasive ventilators or negative pressure ventilators at the initiating stage of care

3. >=17 years of age on the 21^st^ day of mechanical ventilation

4. The date of the 21^st^ day of mechanical ventilation falling in 1998-2007

5. No use of invasive ventilators, negative pressure ventilators, and positive pressure ventilators for at least one year before the first day of this event of prolonged mechanical ventilation

New PMV patients with no AKI and no RRT in the 6 months before the index admission, plus those already requiring permanent need for dialysis before the index admission

(n=47,754)

Those already with permanent need for dialysis before the index admission

(n=1,015)

Those with no AKI and no RRT in the 6 months before the index admission

(n=46,739)

Exclusion criterion:

With acute kidney injury (AKI) or renal replacement therapy (RRT) in the 6 months before the index admission with PMV, *except* those requiring permanent need for dialysis before the index admission for PMV (n=2,727; 5.40%)

Patients who ever used invasive or non-invasive respiratory care in the National Health Insurance (NHI) in 1996-2007 (n=8,906,406; **34%** of all enrolees ever alive in the 12 years)

Those with AKI-dialysis in the index admission

(n=5,129)

Those without AKI/with non AKI-dialysis in the index admission

(n=41,610)

Figure S1. Flow diagram of the selection process of the study subjects
