# Supplementary material for: The Impact of Dialysis-Requiring Acute Kidney Injury on Long-Term Prognosis of Patients Requiring Prolonged Mechanical Ventilation: Nationwide Population-Based Study
Source: PLoS One. 2012 Dec 12;7(12):e50675. doi: 10.1371/journal.pone.0050675 (PMC3520952; doi:10.1371/journal.pone.0050675)
Supplement: Table S1 — Adjusted odds ratios – estimates based on random-effects logistic regression results for mortality. Part 1: In-hospital, 3-month past PMV, and 6-month post-PMV mortality rates. Part 1A: In-hospital, 3-month past PMV, and 6-month post-PMV mortality rates: sensitivity analysis using the ESRD group as the reference group. Part 2A: 1-year, 2-year, 3-year and 4-year past PMV mortality rates: sensitivity analysis using the ESRD group prior to PMV as the reference group. (DOC) [file pone.0050675.s003.doc]

We used random-effects logistic model. The analysis was done using Stata software version 9 (StataCorp, College Station, TX). The model adopted an integration method based on adaptive Gauss-Hermite quadrature. The model can estimate a statistic (ρ) that shows the proportion of the total variance contributed by heterogeneity among individual hospitals. Regarding covariates selected in the logistic model, we referred to literature and data availability to construct a set of potential explanatory variables. We first used stepwise procedure to choose explanatory variables that were suitable for stepwise selection (such as a continuous variable, or a dummy variable alone for a specific feature), and found that almost all potential variables were chosen. Furthermore, results based on stepwise procedure were very close to those based on inclusion of all potential variables. We finally reported model results with all potential variables.

Our main analysis focused on comparison between patients with AKI-dialysis in the index admission and their counterparts with no AKI or non dialysis-requiring AKI. For sensitivity analysis, we also compared patients with AKI-dialysis and those with ESRD. We reported results from main analysis in Parts 1 and 2 of Appendix Table 1, and results from sensitivity analysis in Parts 1A and 1B. Statistically, when we selected an alternative group as the reference group, only coefficients corresponding to the specific set of explanatory variables would change. The other coefficients and statistics regarding model performance would remain the same. Thus, we only reported new results with respect to patient categorization in Parts 1A and 1B.

As indicated by the results (see below), the proportion of total variance contributed by heterogeneity among individual hospitals was quite small (1%~ 6%). In other words, after all covariates were adjusted for, the level of heterogeneity among hospitals was very limited. The results also show that these logistic models had satisfactory performance, as shown by test statistics for model significance.

**Table S1. *Adjusted odds ratios* ^†^ *- estimates based on random-effects logistic regression results for mortality***

Part 1: In-hospital, 3-month past PMV, and 6-month post-PMV mortality rates

|  | in-hospital mortality | | | | |  | 3-month post-PMV mortality | | | | |  | 6-month post-PMV mortality | | | | |
| --- | --- | --- | --- | --- | --- | --- | --- | --- | --- | --- | --- | --- | --- | --- | --- | --- | --- |
| Factor | OR | [95% CI] | | | P>|z| |  | OR | [95% CI] | | | P>|z| |  | OR | [95% CI] | | | P>|z| |
| *Patient group (reference: non-AKI group^††^)* | | | | | | | | | | | | | | | | | |
| AKI-dialysis in index admission^††^ | 2.07 | 1.93 | - | 2.21 | 0.000 |  | 1.53 | 1.43 | - | 1.64 | 0.000 |  | 2.06 | 1.91 | - | 2.22 | 0.000 |
| ESRD prior to PMV | 1.11 | 0.96 | - | 1.27 | 0.166 |  | 1.01 | 0.88 | - | 1.16 | 0.907 |  | 1.38 | 1.18 | - | 1.61 | 0.000 |
| *Male (relative to female)* | 1.11 | 1.06 | - | 1.16 | 0.000 |  | 1.13 | 1.09 | - | 1.18 | 0.000 |  | 1.20 | 1.15 | - | 1.25 | 0.000 |
| *Age group (reference: <45)* |  |  |  |  |  |  |  |  |  |  |  |  |  |  |  |  |  |
| 45-64 | 1.29 | 1.17 | - | 1.43 | 0.000 |  | 1.45 | 1.32 | - | 1.60 | 0.000 |  | 1.57 | 1.43 | - | 1.73 | 0.000 |
| 65-74 | 1.58 | 1.43 | - | 1.75 | 0.000 |  | 1.85 | 1.68 | - | 2.04 | 0.000 |  | 2.22 | 2.02 | - | 2.45 | 0.000 |
| >74 | 1.93 | 1.75 | - | 2.12 | 0.000 |  | 2.22 | 2.02 | - | 2.44 | 0.000 |  | 3.04 | 2.78 | - | 3.33 | 0.000 |
| *ED admission (relative to non-ED admission)* | 0.90 | 0.86 | - | 0.94 | 0.000 |  | 1.06 | 1.01 | - | 1.10 | 0.009 |  | 1.01 | 0.97 | - | 1.06 | 0.548 |
| *ICU admission (relative to non-ICU admission)* | 1.06 | 0.97 | - | 1.15 | 0.210 |  | 1.18 | 1.08 | - | 1.27 | 0.000 |  | 1.18 | 1.09 | - | 1.28 | 0.000 |
| *Number of organ dysfunction during the index admission (excluding lungs and kidneys; reference: zero)* | | | | | | | | | | |  |  |  |  |  |  |  |
| 1 | 1.23 | 1.14 | - | 1.32 | 0.000 |  | 1.04 | 0.97 | - | 1.12 | 0.215 |  | 1.13 | 1.05 | - | 1.21 | 0.001 |
| 2 | 2.47 | 2.28 | - | 2.68 | 0.000 |  | 1.63 | 1.51 | - | 1.76 | 0.000 |  | 1.79 | 1.66 | - | 1.93 | 0.000 |
| 3 | 3.64 | 3.25 | - | 4.07 | 0.000 |  | 2.02 | 1.81 | - | 2.24 | 0.000 |  | 2.14 | 1.91 | - | 2.40 | 0.000 |
| >4 | 3.46 | 2.61 | - | 4.58 | 0.000 |  | 1.47 | 1.12 | - | 1.93 | 0.006 |  | 1.77 | 1.31 | - | 2.38 | 0.000 |
| *Comorbidity during the year prior to the index admission (relative to those without the disease)* | | | | | | | | |  |  |  |  |  |  |  |  |  |
| Parkinson’s disease | 0.87 | 0.76 | - | 0.99 | 0.032 |  | 0.92 | 0.81 | - | 1.04 | 0.183 |  | 0.89 | 0.79 | - | 1.02 | 0.084 |
| MS or degenerative nervous system | 0.91 | 0.78 | - | 1.06 | 0.234 |  | 0.79 | 0.68 | - | 0.93 | 0.003 |  | 0.87 | 0.75 | - | 1.01 | 0.062 |
| Neurologic | 0.94 | 0.90 | - | 0.98 | 0.008 |  | 0.89 | 0.85 | - | 0.93 | 0.000 |  | 0.87 | 0.83 | - | 0.91 | 0.000 |
| Cardiovascular | 0.88 | 0.83 | - | 0.94 | 0.000 |  | 0.88 | 0.83 | - | 0.93 | 0.000 |  | 0.90 | 0.85 | - | 0.96 | 0.001 |
| Pulmonary | 1.01 | 0.96 | - | 1.06 | 0.805 |  | 1.04 | 0.99 | - | 1.09 | 0.167 |  | 1.03 | 0.98 | - | 1.09 | 0.182 |
| COPD | 0.91 | 0.85 | - | 0.97 | 0.002 |  | 0.92 | 0.87 | - | 0.98 | 0.007 |  | 0.90 | 0.85 | - | 0.96 | 0.001 |
| Renal | 1.02 | 0.97 | - | 1.07 | 0.523 |  | 1.00 | 0.95 | - | 1.05 | 0.935 |  | 1.05 | 1.002 | - | 1.11 | 0.041 |
| Hepatic | 1.27 | 1.19 | - | 1.36 | 0.000 |  | 1.34 | 1.26 | - | 1.44 | 0.000 |  | 1.30 | 1.22 | - | 1.40 | 0.000 |
| Cancer | 2.04 | 1.89 | - | 2.21 | 0.000 |  | 1.99 | 1.85 | - | 2.15 | 0.000 |  | 2.27 | 2.08 | - | 2.47 | 0.000 |
| Diabetes | 0.97 | 0.93 | - | 1.03 | 0.322 |  | 1.01 | 0.96 | - | 1.06 | 0.605 |  | 1.10 | 1.05 | - | 1.16 | 0.000 |
| Hypertension | 0.93 | 0.89 | - | 0.98 | 0.011 |  | 0.92 | 0.88 | - | 0.97 | 0.002 |  | 0.92 | 0.87 | - | 0.97 | 0.001 |
| *Charlson index* | 1.07 | 1.06 | - | 1.09 | 0.000 |  | 1.09 | 1.08 | - | 1.11 | 0.000 |  | 1.12 | 1.10 | - | 1.14 | 0.000 |
| *Major operation during the year prior to the index admission (relative to those without such operation)* | | | | | | | | | | |  |  |  |  |  |  |  |
| Cardiac / thoracic aorta | 1.09 | 0.94 | - | 1.27 | 0.241 |  | 1.07 | 0.92 | - | 1.23 | 0.391 |  | 1.14 | 0.98 | - | 1.34 | 0.098 |
| Liver / bililary / pancreas | 0.97 | 0.84 | - | 1.11 | 0.621 |  | 0.93 | 0.82 | - | 1.06 | 0.296 |  | 0.87 | 0.76 | - | 1.00 | 0.052 |
| Lower digestive tract | 1.04 | 0.90 | - | 1.20 | 0.565 |  | 0.97 | 0.84 | - | 1.12 | 0.698 |  | 1.05 | 0.89 | - | 1.22 | 0.577 |
| Upper digestive tract | 1.20 | 1.03 | - | 1.39 | 0.017 |  | 1.11 | 0.96 | - | 1.28 | 0.175 |  | 1.20 | 1.02 | - | 1.41 | 0.029 |
| *Number of days with inpatient care during the year prior to the index admission* | 1.00 | 1.00 | - | 1.00 | 0.000 |  | 1.00 | 1.00 | - | 1.00 | 0.002 |  | 1.0003 | 0.9997 | - | 1.001 | 0.356 |
| *Number of chest films taken during the year prior to the index admission* | 1.12 | 1.02 | - | 1.23 | 0.016 |  | 1.18 | 1.07 | - | 1.29 | 0.000 |  | 1.13 | 1.02 | - | 1.25 | 0.019 |
| *Number of outpatients visits due to lung diseases during the year prior to the index admission* | 1.00 | 1.00 | - | 1.00 | 0.093 |  | 1.00 | 1.00 | - | 1.00 | 0.930 |  | 1.001 | 0.998 | - | 1.003 | 0.607 |
| *ρ (the proportion of the total variance contributed by heterogeneity among individual hospitals)* | *ρ=*  0.06 | 0.05 | - | 0.07 |  |  | *ρ=*  0.02 | 0.01 | - | 0.02 |  |  | *ρ=*  0.02 | 0.02 | - | 0.03 |  |
| Number of individual hospitals (clusters) | 2357 | | | |  |  | 2354 | | | |  |  | 2341 | | | |  |
| Number of observations | 47754 | | | |  |  | 46896 | | | |  |  | 45474 | | | |  |
| Log likelihood | -29116.36 | | | |  |  | -29917.12 | | | |  |  | -28926.35 | | | |  |
| Test statistic for model significance  (Waldχ^2^(31)) | 3866.56 (p-value < 0.001) | | | |  |  | 2805.37 (p-value < 0.001) | | | |  |  | 3865.01 (p-value < 0.001) | | | |  |

Part 1A: In-hospital. 3-month past PMV, and 6-month post-PMV mortality rates: sensitivity analysis using the ESRD group

as the reference group

|  | in-hospital mortality | | | | |  | 3-month post-PMV mortality | | | | |  | 6-month post-PMV mortality | | | | |
| --- | --- | --- | --- | --- | --- | --- | --- | --- | --- | --- | --- | --- | --- | --- | --- | --- | --- |
| Factor | OR | [95% CI] | | | P>|z| |  | OR | [95% CI] | | | P>|z| |  | OR | [95% CI] | | | P>|z| |
| *Patient group (reference: ESRD prior to PMV)* | | | | | | | | | | | | | | | | | |
| AKI-dialysis in index admission†† | 1.87 | 1.61 | - | 2.17 | 0.000 |  | 1.52 | 1.31 | - | 1.76 | 0.000 |  | 1.50 | 1.27 | - | 1.76 | 0.000 |
| Non-AKI†† | 0.90 | 0.78 | - | 1.04 | 0.166 |  | 0.99 | 0.86 | - | 1.14 | 0.907 |  | 0.73 | 0.62 | - | 0.85 | 0.000 |

Part 2: 1-year, 2-year, 3-year and 4-year past PMV mortality rates

|  | 1-year post-PMV mortality | | | | |  | 2-year post-PMV mortality | | | | |  | 3-year post-PMV mortality | | | | |  | 4-year post-PMV mortality | | | | |
| --- | --- | --- | --- | --- | --- | --- | --- | --- | --- | --- | --- | --- | --- | --- | --- | --- | --- | --- | --- | --- | --- | --- | --- |
| Factor | OR | [95% CI] | | | P>|z| |  | OR | [95% CI] | | | P>|z| |  | OR | [95% CI] | | | P>|z| |  | OR | [95% CI] | | | P>|z| |
| *Patient group (reference: non-AKI group in index admission^††^)* | | | | | | | | | | | | | | | | | |  |  |  |  |  |  |
| AKI-dialysis in index admission^††^ | 2.24 | 2.06 | - | 2.45 | 0.000 |  | 2.10 | 1.89 | - | 2.34 | 0.000 |  | 2.02 | 1.79 | - | 2.30 | 0.000 |  | 1.89 | 1.63 | - | 2.19 | 0.000 |
| ESRD prior to PMV | 1.57 | 1.29 | - | 1.90 | 0.000 |  | 1.31 | 1.01 | - | 1.69 | 0.041 |  | 0.88 | 0.64 | - | 1.22 | 0.452 |  | 0.50 | 0.33 | - | 0.77 | 0.001 |
| *Male (relative to female)* | 1.26 | 1.20 | - | 1.31 | 0.000 |  | 1.28 | 1.22 | - | 1.35 | 0.000 |  | 1.26 | 1.19 | - | 1.34 | 0.000 |  | 1.22 | 1.14 | - | 1.31 | 0.000 |
| *Age group (reference: <45)* |  |  |  |  |  |  |  |  |  |  |  |  |  |  |  |  |  |  |  |  |  |  |  |
| 45-64 | 1.66 | 1.51 | - | 1.84 | 0.000 |  | 1.81 | 1.63 | - | 2.01 | 0.000 |  | 1.92 | 1.71 | - | 2.15 | 0.000 |  | 1.87 | 1.64 | - | 2.13 | 0.000 |
| 65-74 | 2.63 | 2.38 | - | 2.90 | 0.000 |  | 3.07 | 2.76 | - | 3.41 | 0.000 |  | 3.35 | 2.98 | - | 3.76 | 0.000 |  | 3.48 | 3.05 | - | 3.97 | 0.000 |
| >74 | 3.93 | 3.57 | - | 4.31 | 0.000 |  | 4.83 | 4.37 | - | 5.35 | 0.000 |  | 5.36 | 4.79 | - | 6.00 | 0.000 |  | 5.73 | 5.05 | - | 6.50 | 0.000 |
| *ED admission (relative to non-ED admission)* | 1.04 | 0.99 | - | 1.09 | 0.145 |  | 1.05 | 0.99 | - | 1.11 | 0.120 |  | 1.01 | 0.95 | - | 1.08 | 0.754 |  | 1.02 | 0.95 | - | 1.10 | 0.606 |
| *ICU admission (relative to non-ICU admission)* | 1.24 | 1.14 | - | 1.35 | 0.000 |  | 1.31 | 1.19 | - | 1.44 | 0.000 |  | 1.34 | 1.20 | - | 1.50 | 0.000 |  | 1.38 | 1.21 | - | 1.57 | 0.000 |
| *Number of organ dysfunction during the index admission (excluding lungs and kidneys; reference: zero)* | | | | | | | | | | |  |  |  |  |  |  |  |  |  |  |  |  |  |
| 1 | 1.26 | 1.17 | - | 1.35 | 0.000 |  | 1.25 | 1.15 | - | 1.35 | 0.000 |  | 1.31 | 1.21 | - | 1.43 | 0.000 |  | 1.31 | 1.19 | - | 1.43 | 0.000 |
| 2 | 1.95 | 1.79 | - | 2.11 | 0.000 |  | 1.88 | 1.72 | - | 2.07 | 0.000 |  | 1.88 | 1.69 | - | 2.08 | 0.000 |  | 1.79 | 1.59 | - | 2.02 | 0.000 |
| 3 | 2.37 | 2.08 | - | 2.70 | 0.000 |  | 2.14 | 1.83 | - | 2.51 | 0.000 |  | 2.13 | 1.77 | - | 2.57 | 0.000 |  | 1.81 | 1.46 | - | 2.24 | 0.000 |
| >4 | 2.37 | 1.64 | - | 3.42 | 0.000 |  | 2.72 | 1.68 | - | 4.40 | 0.000 |  | 2.81 | 1.54 | - | 5.11 | 0.001 |  | 1.84 | 0.92 | - | 3.65 | 0.084 |
| *Comorbidity during the year prior to the index admission (relative to those without the disease)* | | | | | | | | |  |  |  |  |  |  |  |  |  |  |  |  |  |  |  |
| Parkinson’s disease | 0.90 | 0.78 | - | 1.03 | 0.125 |  | 1.03 | 0.87 | - | 1.23 | 0.711 |  | 1.22 | 0.98 | - | 1.53 | 0.076 |  | 1.32 | 0.99 | - | 1.75 | 0.055 |
| MS or degenerative nervous system | 0.93 | 0.79 | - | 1.09 | 0.385 |  | 0.83 | 0.69 | - | 1.00 | 0.048 |  | 0.95 | 0.76 | - | 1.20 | 0.687 |  | 0.94 | 0.71 | - | 1.24 | 0.656 |
| Neurologic | 0.87 | 0.82 | - | 0.91 | 0.000 |  | 0.89 | 0.84 | - | 0.95 | 0.000 |  | 0.91 | 0.85 | - | 0.98 | 0.013 |  | 0.95 | 0.87 | - | 1.03 | 0.203 |
| Cardiovascular | 0.94 | 0.88 | - | 1.00 | 0.041 |  | 1.03 | 0.96 | - | 1.11 | 0.373 |  | 1.05 | 0.96 | - | 1.14 | 0.255 |  | 1.08 | 0.98 | - | 1.19 | 0.143 |
| Pulmonary | 1.06 | 1.01 | - | 1.12 | 0.032 |  | 1.07 | 1.004 | - | 1.14 | 0.036 |  | 1.07 | 1.00 | - | 1.16 | 0.062 |  | 1.07 | 0.98 | - | 1.17 | 0.133 |
| COPD | 0.93 | 0.86 | - | 0.99 | 0.026 |  | 0.96 | 0.89 | - | 1.05 | 0.369 |  | 1.004 | 0.91 | - | 1.11 | 0.943 |  | 1.005 | 0.89 | - | 1.14 | 0.939 |
| Renal | 1.12 | 1.06 | - | 1.19 | 0.000 |  | 1.16 | 1.09 | - | 1.25 | 0.000 |  | 1.18 | 1.08 | - | 1.28 | 0.000 |  | 1.15 | 1.05 | - | 1.27 | 0.004 |
| Hepatic | 1.23 | 1.13 | - | 1.33 | 0.000 |  | 1.19 | 1.08 | - | 1.31 | 0.000 |  | 1.13 | 1.01 | - | 1.27 | 0.031 |  | 1.12 | 0.98 | - | 1.28 | 0.107 |
| Cancer | 2.44 | 2.21 | - | 2.70 | 0.000 |  | 2.47 | 2.17 | - | 2.80 | 0.000 |  | 2.30 | 1.97 | - | 2.69 | 0.000 |  | 2.24 | 1.86 | - | 2.70 | 0.000 |
| Diabetes | 1.15 | 1.09 | - | 1.22 | 0.000 |  | 1.22 | 1.13 | - | 1.30 | 0.000 |  | 1.24 | 1.14 | - | 1.35 | 0.000 |  | 1.26 | 1.14 | - | 1.40 | 0.000 |
| Hypertension | 0.95 | 0.90 | - | 1.01 | 0.102 |  | 0.97 | 0.91 | - | 1.03 | 0.340 |  | 0.99 | 0.92 | - | 1.08 | 0.882 |  | 1.05 | 0.96 | - | 1.15 | 0.285 |
| *Charlson index* | 1.14 | 1.12 | - | 1.16 | 0.000 |  | 1.16 | 1.13 | - | 1.18 | 0.000 |  | 1.17 | 1.14 | - | 1.20 | 0.000 |  | 1.18 | 1.14 | - | 1.22 | 0.000 |
| *Major operation during the year prior to the index admission (relative to those without such operation)* | | | | | | | | | | |  |  |  |  |  |  |  |  |  |  |  |  |  |
| Cardiac / thoracic aorta | 1.29 | 1.07 | - | 1.57 | 0.009 |  | 1.48 | 1.15 | - | 1.92 | 0.003 |  | 1.51 | 1.09 | - | 2.08 | 0.012 |  | 1.40 | 0.94 | - | 2.07 | 0.094 |
| Liver / bililary / pancrease | 0.82 | 0.70 | - | 0.96 | 0.014 |  | 0.83 | 0.68 | - | 1.01 | 0.070 |  | 0.80 | 0.63 | - | 1.02 | 0.066 |  | 0.73 | 0.54 | - | 0.98 | 0.036 |
| Lower digestive tract | 1.04 | 0.86 | - | 1.25 | 0.691 |  | 0.99 | 0.78 | - | 1.25 | 0.911 |  | 0.98 | 0.73 | - | 1.31 | 0.904 |  | 1.04 | 0.71 | - | 1.51 | 0.839 |
| Upper digestive tract | 1.39 | 1.15 | - | 1.69 | 0.001 |  | 1.16 | 0.92 | - | 1.47 | 0.215 |  | 1.07 | 0.80 | - | 1.41 | 0.660 |  | 1.11 | 0.79 | - | 1.56 | 0.559 |
| *Number of days with inpatient care during the year prior to the index admission* | 1.001 | 1.0001 | - | 1.001 | 0.015 |  | 1.002 | 1.001 | - | 1.002 | 0.000 |  | 1.002 | 1.001 | - | 1.003 | 0.000 |  | 1.002 | 1.001 | - | 1.003 | 0.001 |
| *Number of chest films taken during the year prior to the index admission* | 1.12 | 0.99 | - | 1.26 | 0.063 |  | 1.06 | 0.91 | - | 1.23 | 0.462 |  | 1.01 | 0.84 | - | 1.22 | 0.924 |  | 0.93 | 0.75 | - | 1.15 | 0.505 |
| *Number of outpatients visits due to lung diseases during the year prior to the index admission* | 0.999 | 0.997 | - | 1.002 | 0.563 |  | 1.000 | 0.997 | - | 1.003 | 0.819 |  | 1.002 | 0.999 | - | 1.01 | 0.243 |  | 1.01 | 1.001 | - | 1.01 | 0.016 |
| *ρ (the proportion of the total variance contributed by heterogeneity among individual hospitals)* | *ρ=*  0.03 | 0.02 | - | 0.03 |  |  | *ρ=*  0.02 | 0.02 | - | 0.03 |  |  | *ρ=*  0.02 | 0.01 | - | 0.03 |  |  | *ρ=*  0.01 | 0.01 | - | 0.02 |  |
| Number of individual hospitals (clusters) | 2119 | | | |  |  | 1867 | | | |  |  | 1618 | | | |  |  | 1343 | | | |  |
| Number of observations | 42333 | | | |  |  | 36616 | | | |  |  | 30510 | | | |  |  | 24436 | | | |  |
| Log likelihood | -24699.16 | | | |  |  | -18655.17 | | | |  |  | -13983.77 | | | |  |  | -10259.65 | | | |  |
| Test statistic for model significance  (Wald χ^2^(31)) | 4052.66 (p-value < 0.001) | | | |  |  | 3505.49 (p-value < 0.001) | | | |  |  | 2874.62 (p-value < 0.001) | | | |  |  | 2263.44 (p-value < 0.001) | | | |  |

Part 2A: 1-year, 2-year, 3-year and 4-year past PMV mortality rates: sensitivity analysis using the ESRD group prior to PMV as the reference group

|  | 1-year post-PMV mortality | | | | |  | 2-year post-PMV mortality | | | | |  | 3-year post-PMV mortality | | | | |  | 4-year post-PMV mortality | | | | |
| --- | --- | --- | --- | --- | --- | --- | --- | --- | --- | --- | --- | --- | --- | --- | --- | --- | --- | --- | --- | --- | --- | --- | --- |
| Factor | OR | [95% CI] | | | P>|z| |  | OR | [95% CI] | | | P>|z| |  | OR | [95% CI] | | | P>|z| |  | OR | [95% CI] | | | P>|z| |
| *Patient group (reference: ESRD prior to PMV)* | | | | | | | | | | | | | | | | | |  |  |  |  |  |  |
| AKI-dialysis in index admission†† | 1.43 | 1.17 | - | 1.76 | 0.001 |  | 1.61 | 1.23 | - | 2.10 | 0.001 |  | 2.29 | 1.64 | - | 3.20 | 0.000 |  | 3.75 | 2.43 | - | 5.81 | 0.000 |
| Non-AKI in index admission†† | 0.64 | 0.53 | - | 0.78 | 0.000 |  | 0.76 | 0.59 | - | 0.99 | 0.041 |  | 1.13 | 0.82 | - | 1.56 | 0.452 |  | 1.98 | 1.30 | - | 3.03 | 0.001 |

Abbreviations: AKI, acute kidney injury; CI, confidence interval; COPD, chronic obstructive pulmonary disease; ED, emergency department; ESRD, end-stage renal disease; HR, hazards ratio; ICU, intensive care unit; MS, multiple sclerosis; OR, odds ratio; PMV, prolonged mechanical ventilation; RRT, renal replacement therapy

^†^ All other covariates were controlled for.

^††^ No AKI and no RRT prior to PMV
