# Supplementary material for: The Impact of Dialysis-Requiring Acute Kidney Injury on Long-Term Prognosis of Patients Requiring Prolonged Mechanical Ventilation: Nationwide Population-Based Study
Source: PLoS One. 2012 Dec 12;7(12):e50675. doi: 10.1371/journal.pone.0050675 (PMC3520952; doi:10.1371/journal.pone.0050675)
Supplement: Table S2 — Adjusted relative risk ratios – estimates based on random-effects logistic regression results for mortality. Part 1: Main analysis using patients without AKI-dialysis as the reference patient group. Part 2: sensitivity analysis using patients with ESRD as the reference patient group. (DOC) [file pone.0050675.s004.doc]

**Table S2. *Adjusted relative risk ratios*^†^ *- estimates based on random-effects logistic regression results for mortality***

Part 1: Main analysis using patients without AKI-dialysis as the reference patient group

|  | In-hospital mortality | | | 3-month post-PMV mortality | | | 6-month post-PMV mortality | | | 1-year post-PMV mortality | | | 2-year post-PMV mortality | | | 3-year post-PMV mortality | | | 4-year post-PMV mortality | | |
| --- | --- | --- | --- | --- | --- | --- | --- | --- | --- | --- | --- | --- | --- | --- | --- | --- | --- | --- | --- | --- | --- |
|  | ARRR | 95% CI | | ARRR | 95% CI | | ARRR | 95% CI | | ARRR | 95% CI | | ARRR | 95% CI | | ARRR | 95% CI | | ARRR | 95% CI | |
| *Patient group (reference: Non-AKI during index admission^††^)* | | | | | | | | | | | | | | | | | | | | | |
| AKI-dialysis during index admission^††^ | 1.51 | 1.46 | -1.56 | 1.27 | 1.23 | -1.32 | 1.33 | 1.30 | -1.36 | 1.26 | 1.24 | -1.28 | 1.17 | 1.15 | -1.19 | 1.13 | 1.11 | -1.15 | 1.10 | 1.08 | -1.12 |
| ESRD prior to PMV | 1.07 | 0.97 | -1.16 | 1.01 | 0.92 | -1.09 | 1.15 | 1.08 | -1.22 | 1.16 | 1.09 | -1.21 | 1.07 | 1.00 | -1.13 | 0.97 | 0.89 | -1.04 | 0.84 | 0.72 | -0.95 |
| *Male (relative to female)* | 1.05 | 1.03 | -1.08 | 1.07 | 1.05 | -1.09 | 1.05 | 1.04 | -1.07 | 1.04 | 1.03 | -1.05 | 1.03 | 1.03 | -1.04 | 1.03 | 1.03 | -1.04 | 1.04 | 1.03 | -1.05 |
| *Age group (reference: <45)* | | | | | | | | | | | | | | | | | | | | | |
| 45-64 | 1.13 | 1.08 | -1.19 | 1.20 | 1.15 | -1.26 | 1.13 | 1.10 | -1.15 | 1.08 | 1.07 | -1.10 | 1.07 | 1.06 | -1.08 | 1.08 | 1.07 | -1.09 | 1.11 | 1.09 | -1.13 |
| 65-74 | 1.24 | 1.19 | -1.29 | 1.33 | 1.28 | -1.38 | 1.21 | 1.19 | -1.23 | 1.14 | 1.13 | -1.14 | 1.11 | 1.10 | -1.11 | 1.12 | 1.12 | -1.13 | 1.18 | 1.17 | -1.19 |
| >74 | 1.34 | 1.29 | -1.38 | 1.42 | 1.38 | -1.47 | 1.27 | 1.25 | -1.28 | 1.17 | 1.16 | -1.17 | 1.13 | 1.12 | -1.13 | 1.14 | 1.14 | -1.15 | 1.21 | 1.20 | -1.22 |
| *ED admission (relative to non-ED admission)* | 0.94 | 0.92 | -0.97 | 1.03 | 1.01 | -1.05 | 1.00 | 0.99 | -1.02 | 1.01 | 1.00 | -1.02 | 1.01 | 1.00 | -1.01 | 1.00 | 0.99 | -1.01 | 1.00 | 0.99 | -1.02 |
| *ICU admission (relative to non-ICU admission)* | 1.03 | 0.98 | -1.07 | 1.09 | 1.04 | -1.13 | 1.05 | 1.03 | -1.07 | 1.04 | 1.02 | -1.05 | 1.03 | 1.02 | -1.05 | 1.04 | 1.03 | -1.05 | 1.06 | 1.04 | -1.08 |
| *Number of organ dysfunction during the index admission (excluding lungs and kidneys; reference: zero)* | | | | | | | | | | | | | | | | | | | | | |
| 1 | 1.11 | 1.07 | -1.15 | 1.02 | 0.99 | -1.06 | 1.04 | 1.02 | -1.06 | 1.04 | 1.03 | -1.05 | 1.03 | 1.02 | -1.04 | 1.04 | 1.03 | -1.05 | 1.05 | 1.03 | -1.07 |
| 2 | 1.46 | 1.42 | -1.49 | 1.26 | 1.22 | -1.30 | 1.16 | 1.14 | -1.18 | 1.10 | 1.09 | -1.11 | 1.07 | 1.06 | -1.08 | 1.08 | 1.07 | -1.09 | 1.10 | 1.09 | -1.12 |
| 3 | 1.62 | 1.57 | -1.66 | 1.38 | 1.32 | -1.43 | 1.20 | 1.18 | -1.23 | 1.12 | 1.11 | -1.14 | 1.08 | 1.07 | -1.09 | 1.09 | 1.07 | -1.10 | 1.10 | 1.07 | -1.13 |
| >4 | 1.60 | 1.48 | -1.70 | 1.21 | 1.06 | -1.35 | 1.16 | 1.08 | -1.22 | 1.12 | 1.08 | -1.16 | 1.10 | 1.06 | -1.12 | 1.11 | 1.06 | -1.14 | 1.11 | 0.98 | -1.18 |
| *Comorbidity during the year prior to the index admission (relative to those without the disease)* | | | | | | | | | | | | | | | | | | | | | |
| Parkinson’s disease | 0.93 | 0.86 | -0.99 | 0.95 | 0.89 | -1.02 | 0.96 | 0.92 | -1.00 | 0.98 | 0.95 | -1.01 | 1.00 | 0.98 | -1.03 | 1.03 | 1.00 | -1.06 | 1.05 | 1.00 | -1.10 |
| MS or degenerative nervous system | 0.95 | 0.87 | -1.03 | 0.88 | 0.80 | -0.96 | 0.95 | 0.90 | -1.00 | 0.99 | 0.95 | -1.02 | 0.97 | 0.94 | -1.00 | 0.99 | 0.95 | -1.03 | 0.99 | 0.92 | -1.04 |
| Neurologic | 0.97 | 0.94 | -1.00 | 0.94 | 0.91 | -0.96 | 0.95 | 0.94 | -0.97 | 0.97 | 0.96 | -0.98 | 0.98 | 0.97 | -0.99 | 0.99 | 0.97 | -1.00 | 0.99 | 0.97 | -1.01 |
| Cardiovascular | 0.93 | 0.90 | -0.97 | 0.93 | 0.90 | -0.96 | 0.97 | 0.95 | -0.99 | 0.99 | 0.97 | -1.00 | 1.00 | 0.99 | -1.01 | 1.01 | 0.99 | -1.02 | 1.02 | 0.99 | -1.03 |
| Pulmonary | 1.00 | 0.98 | -1.03 | 1.02 | 0.99 | -1.05 | 1.01 | 0.99 | -1.03 | 1.01 | 1.00 | -1.02 | 1.01 | 1.00 | -1.02 | 1.01 | 1.00 | -1.02 | 1.01 | 1.00 | -1.03 |
| COPD | 0.95 | 0.92 | -0.98 | 0.96 | 0.92 | -0.99 | 0.97 | 0.95 | -0.99 | 0.98 | 0.97 | -1.00 | 0.99 | 0.98 | -1.01 | 1.00 | 0.98 | -1.02 | 1.00 | 0.97 | -1.03 |
| Renal | 1.01 | 0.98 | -1.03 | 1.00 | 0.97 | -1.03 | 1.02 | 1.00 | -1.03 | 1.02 | 1.01 | -1.03 | 1.02 | 1.01 | -1.03 | 1.02 | 1.01 | -1.03 | 1.03 | 1.01 | -1.05 |
| Hepatic | 1.13 | 1.09 | -1.16 | 1.16 | 1.13 | -1.20 | 1.08 | 1.06 | -1.10 | 1.04 | 1.02 | -1.05 | 1.02 | 1.01 | -1.03 | 1.02 | 1.00 | -1.03 | 1.02 | 0.99 | -1.05 |
| Cancer | 1.37 | 1.33 | -1.40 | 1.37 | 1.33 | -1.41 | 1.21 | 1.20 | -1.23 | 1.13 | 1.12 | -1.14 | 1.09 | 1.08 | -1.10 | 1.10 | 1.08 | -1.11 | 1.13 | 1.11 | -1.15 |
| Diabetes | 0.99 | 0.96 | -1.01 | 1.01 | 0.98 | -1.03 | 1.03 | 1.01 | -1.05 | 1.03 | 1.02 | -1.04 | 1.03 | 1.02 | -1.03 | 1.03 | 1.02 | -1.04 | 1.05 | 1.03 | -1.06 |
| Hypertension | 0.96 | 0.94 | -0.99 | 0.96 | 0.93 | -0.98 | 0.97 | 0.96 | -0.99 | 0.99 | 0.98 | -1.00 | 1.00 | 0.99 | -1.00 | 1.00 | 0.99 | -1.01 | 1.01 | 0.99 | -1.03 |
| *Charlson index* | 1.04 | 1.03 | -1.04 | 1.05 | 1.04 | -1.05 | 1.03 | 1.03 | -1.04 | 1.02 | 1.02 | -1.03 | 1.02 | 1.02 | -1.02 | 1.02 | 1.02 | -1.03 | 1.03 | 1.03 | -1.04 |
| *Major operation during the year prior to the index admission (relative to those without such operation)* | | | | | | | | | | | | | | | | | | | | | |
| Cardiac / thoracic aorta | 1.05 | 0.97 | -1.13 | 1.03 | 0.96 | -1.11 | 1.04 | 0.99 | -1.09 | 1.05 | 1.01 | -1.07 | 1.05 | 1.02 | -1.07 | 1.06 | 1.01 | -1.09 | 1.06 | 0.99 | -1.12 |
| Liver / bililary / pancrea | 0.98 | 0.91 | -1.05 | 0.96 | 0.89 | -1.03 | 0.96 | 0.91 | -1.00 | 0.96 | 0.92 | -0.99 | 0.97 | 0.94 | -1.00 | 0.96 | 0.92 | -1.00 | 0.93 | 0.85 | -1.00 |
| Lower digestive tract | 1.02 | 0.95 | -1.10 | 0.98 | 0.91 | -1.06 | 1.01 | 0.96 | -1.06 | 1.01 | 0.97 | -1.04 | 1.00 | 0.96 | -1.03 | 1.00 | 0.95 | -1.04 | 1.01 | 0.92 | -1.08 |
| Upper digestive tract | 1.10 | 1.02 | -1.17 | 1.06 | 0.98 | -1.14 | 1.05 | 1.01 | -1.10 | 1.06 | 1.03 | -1.08 | 1.02 | 0.99 | -1.05 | 1.01 | 0.96 | -1.05 | 1.02 | 0.95 | -1.08 |
| *Number of days with inpatient care during the year prior to the index admission* | 1.00 | 1.00 | -1.00 | 1.00 | 1.00 | -1.00 | 1.00 | 1.00 | -1.00 | 1.00 | 1.00 | -1.00 | 1.00 | 1.00 | -1.00 | 1.00 | 1.00 | -1.00 | 1.00 | 1.00 | -1.00 |
| *Number of chest films taken during the year prior to the index admission* | 1.06 | 1.01 | -1.11 | 1.09 | 1.04 | -1.14 | 1.04 | 1.01 | -1.07 | 1.02 | 1.00 | -1.04 | 1.01 | 0.99 | -1.03 | 1.00 | 0.97 | -1.03 | 0.98 | 0.93 | -1.03 |
| *Number of outpatients visits due to lung diseases during the year prior to the index admission* | 1.00 | 1.00 | -1.00 | 1.00 | 1.00 | -1.00 | 1.00 | 1.00 | -1.00 | 1.00 | 1.00 | -1.00 | 1.00 | 1.00 | -1.00 | 1.00 | 1.00 | -1.00 | 1.00 | 1.00 | -1.00 |

Part 2: Sensitivity analysis using patients with ESRD as the reference patient group

|  | In-hospital mortality | | | 3-month post-PMV mortality | | | 6-month post-PMV mortality | | | 1-year post-PMV mortality | | | 2-year post-PMV mortality | | | 3-year post-PMV mortality | | | 4-year post-PMV mortality | | |
| --- | --- | --- | --- | --- | --- | --- | --- | --- | --- | --- | --- | --- | --- | --- | --- | --- | --- | --- | --- | --- | --- |
|  | ARRR | 95% CI | | ARRR | 95% CI | | ARRR | 95% CI | | ARRR | 95% CI | | ARRR | 95% CI | | ARRR | 95% CI | | ARRR | 95% CI | |
| *Patient group (reference: ESRD prior to PMV)* | | | | | | | | | | | | | | | | | | | | | |
| AKI-dialysis during index admission^††^ | 1.32 | 1.25 | -1.40 | 1.23 | 1.15 | -1.30 | 1.12 | 1.07 | -1.16 | 1.06 | 1.03 | -1.09 | 1.06 | 1.03 | -1.08 | 1.10 | 1.06 | -1.12 | 1.18 | 1.14 | -1.21 |
| Non-AKI during index admission^††^ | 0.95 | 0.87 | -1.02 | 1.00 | 0.92 | -1.07 | 0.89 | 0.84 | -0.95 | 0.90 | 0.85 | -0.95 | 0.96 | 0.91 | -1.00 | 1.02 | 0.97 | -1.06 | 1.12 | 1.05 | -1.17 |

Abbreviations: AKI, acute kidney injury; ARRR, adjusted relative risk ratio; CI, confidence interval; COPD, chronic obstructive pulmonary disease; ED, emergency department; ESRD, end-stage renal disease; HR, hazards ratio; ICU, intensive care unit; MS, multiple sclerosis; PMV, prolonged mechanical ventilation; RRT, renal replacement therapy

^†^ All other covariates were controlled for.

^††^ No AKI and no RRT prior to PMV
