# Supplementary material for: The Impact of Dialysis-Requiring Acute Kidney Injury on Long-Term Prognosis of Patients Requiring Prolonged Mechanical Ventilation: Nationwide Population-Based Study
Source: PLoS One. 2012 Dec 12;7(12):e50675. doi: 10.1371/journal.pone.0050675 (PMC3520952; doi:10.1371/journal.pone.0050675)
Supplement: Table S3 — Sensitivity analysis on the adjusted RRRs of PMV patients with dialysis-requiring AKI for 3-year and 4-year mortality rates. (DOC) [file pone.0050675.s005.doc]

Comparing ESRD and dialysis-requiring AKI, we found that the excess risk of mortality from dialysis-requiring AKI during hospitalization decreased progressively to 23% and 12% at 3 month and 6 months after PMV onset respectively (Appendix Table 2). The excess risk further diminished to less than 10% at 1-year post-PMV but remained significant. The excess risk rose again after 4 years post-PMV. This resurgence of risk might be due to the heterogeneity between ESRD patients receiving PMV care in the early 2000s or earlier and ESRD patients under PMV at a later time. ESRD patients with higher frailty expectedly showed higher mortality, and the corresponding analysis would generate lower adjusted ORs for death when these patients were compared with AKI patients. This implies that the level of excess risk of death could vary with the inherent frailty of ESRD patients selected for comparison.

We further conducted sensitivity analyses on the adjusted ORs and RRRs of 3-year and 4-year post-PMV mortalities under different levels of frailty among ESRD patients. We formulated a few hypothetical scenarios by selecting some mortality rates higher than observed mortality rates for ESRD patients receiving PMV care in Taiwan in 2004 or earlier, who appeared to be more robust than ESRD patients under PMV in later years. Correspondingly, for the hypothetical adjusted OR of AKI relative to ESRD, we selected some values no higher than (equal to or lower than) estimates that we acquired from our previous random-effects logistic regression analyses (since the scenarios were for ESRD patients with higher frailty). With the hypothetical mortality rates for ESRD patients and the hypothetical OR values, we further calculated corresponding RRR values. Results from the sensitivity analyses are shown below.

***Table S3. Sensitivity analysis on the adjusted RRRs† of PMV patients with dialysis-requiring AKI for 3-year and 4-year mortality rates***

| Time of observation | Mortality of ESRD patients (%) | | Adjusted OR (AKI relative to ESRD) | Adjusted RRR (AKI relative to ESRD) |
| --- | --- | --- | --- | --- |
| *Original data* | | | | |
| 3 years after PMV (PMV incidence before 2004) | | 84.44 | 2.29 | 1.10 |
| 4 years after PMV (PMV incidence before 2003) | | 78.85 | 3.75 | 1.18 |
|  |  | |  |  |
| *Hypothetical scenario 1* | | | | |
| 3 years after PMV | 88.00 | | 2.29 | 1.07 |
| 4 years after PMV | 90.00 | | 3.75 | 1.08 |
| *Hypothetical scenario 2* | | | | |
| 3 years after PMV | 88.00 | | 2.10 | 1.07 |
| 4 years after PMV | 90.00 | | 2.80 | 1.07 |
| *Hypothetical scenario 3* | | | | |
| 3 years after PMV | 88.00 | | 1.70 | 1.05 |
| 4 years after PMV | 90.00 | | 1.70 | 1.04 |
| *Hypothetical scenario 4* | | | | |
| 3 years after PMV | 88.00 | | 1.50 | 1.04 |
| 4 years after PMV | 90.00 | | 1.50 | 1.03 |
| *Hypothetical scenario 5* | | | | |
| 3 years after PMV | 90.00 | | 1.50 | 1.03 |
| 4 years after PMV | 93.00 | | 1.50 | 1.02 |

Abbreviations: AKI, acute kidney injury; ESRD, end-stage renal disease; OR, odds ratio; PMV, prolonged mechanical ventilation; RRR, relative risk ratio

^†^ All other covariates were controlled for.
