# Supplementary material for: The Impact of Dialysis-Requiring Acute Kidney Injury on Long-Term Prognosis of Patients Requiring Prolonged Mechanical Ventilation: Nationwide Population-Based Study
Source: PLoS One. 2012 Dec 12;7(12):e50675. doi: 10.1371/journal.pone.0050675 (PMC3520952; doi:10.1371/journal.pone.0050675)
Supplement: Table S4 — Simulated mortality rates (%) for different renal statuses. (DOC) [file pone.0050675.s006.doc]

***Table S4. Simulated mortality rates^†^ (%) for different renal statuses***

|  | Patients for simulation:  all the 3 groups (n= 47754 at the baseline) | | | |  | Patients for simulation:  the original ESRD group (n= 1015 at the baseline) | | | |
| --- | --- | --- | --- | --- | --- | --- | --- | --- | --- |
| follow-up time | sample  size (n) | Everyone  had ESRD | Everyone had  AKI-dialysis | Everyone had  Non-AKI |  | sample  size (n) | Everyone  had ESRD | Everyone had  AKI-dialysis | Everyone had  non-AKI |
| In-hospital | 47754 | 38.7 | 53.0 | 36.5 |  | 1015 | 51.1 | 65.3 | 48.8 |
| 3 months | 46896 | 39.3 | 49.0 | 39.1 |  | 979 | 47.3 | 57.2 | 47.1 |
| 6 months | 45474 | 60.5 | 69.0 | 53.3 |  | 925 | 70.4 | 77.7 | 63.8 |
| 1 year | 42333 | 73.4 | 79.3 | 64.8 |  | 802 | 82.8 | 87.1 | 75.9 |
| 2 years | 36616 | 78.4 | 84.8 | 74.0 |  | 572 | 87.2 | 91.5 | 84.1 |
| 3 years | 30510 | 76.8^††^ | 87.4 | 78.6 |  | 347 | 85.8^††^ | 93.0 | 87.2 |
| 4 years | 24436 | 70.8^††^ | 88.6 | 81.5 |  | 156 | 80.1^††^ | 93.3 | 88.3 |
|  | Patients for simulation:  the original “AKI-dialysis” group (n= 5129 at the baseline) | | | |  | Patients for simulation:  the original “no AKI/non AKI-dialysis” group  (n= 41610 at the baseline) | | | |
| follow-up time | sample  size (n) | Everyone  had ESRD | Everyone had  AKI-dialysis | Everyone had  non-AKI |  | sample  size (n) | Everyone  had ESRD | Everyone had  AKI-dialysis | Everyone had  non-AKI |
| In-hospital | 5129 | 46.9 | 61.2 | 44.5 |  | 41610 | 37.4 | 51.7 | 35.2 |
| 3 months | 5046 | 44.7 | 54.5 | 44.5 |  | 40871 | 38.5 | 48.1 | 38.3 |
| 6 months | 4881 | 66.0 | 73.9 | 59.1 |  | 39668 | 59.6 | 68.2 | 52.4 |
| 1 year | 4561 | 78.0 | 83.2 | 70.1 |  | 36970 | 72.6 | 78.7 | 63.9 |
| 2 years | 3949 | 82.2 | 87.7 | 78.4 |  | 32095 | 77.7 | 84.3 | 73.3 |
| 3 years | 3316 | 80.6^††^ | 89.8 | 82.3 |  | 26847 | 76.2^††^ | 87.0 | 78.1 |
| 4 years | 2664 | 74.6^††^ | 90.5 | 84.3 |  | 21616 | 70.2^††^ | 88.3 | 81.1 |

^†^ For each single patient, except renal status, all other covariates were kept at the original values.

^††^ ESRD patients allowed for PMV care use before 2004 had particularly high robustness, thus we omitted these simulated points in Figure 1, where we depicted the results using curves.

Abbreviations: AKI, acute kidney injury; ESRD, end-stage renal disease.
